# Draft genome sequence of *Xanthomonas arboricola* pv. pruni PVCT 262.1 isolated from *Prunus dulcis* in italy

**DOI:** 10.1128/mra.00273-24

**Published:** 2024-06-11

**Authors:** Giulio Dimaria, Alexandros Mosca, Marcella Russo, Jaime Cubero, Joël F. Pothier, Ralf Koebnik, Vittoria Catara

**Affiliations:** 1 Department of Agriculture, Food, and Environment, University of Catania, Catania, Italy; 2 Agrobiotech Soc. Coop., Catania, Italy; 3 Departamento de Protección Vegetal, Instituto Nacional de Investigación y Tecnología Agraria y Alimentaria/Consejo Superior de Investigaciones Científicas (INIA/CSIC), Madrid, Spain; 4 Environmental Genomics and Systems Biology Research Group, Institute for Natural Resource Sciences, Zurich University of Applied Sciences (ZHAW), Wädenswil, Switzerland; 5 Plant Health Institute of Montpellier, University of Montpellier, CIRAD, INRAE, Institut Agro, IRD, Montpellier, France; The University of Arizona, Tucson, Arizona, USA

**Keywords:** *Xanthomonas*, plant pathology, genomics, *Prunus*

## Abstract

Here, we report the draft genome sequence of *Xanthomonas arboricola* pv. pruni strain PVCT 262.1, isolated from almond (*Prunus dulcis*) leaves affected by bacterial spots in Italy in 2020. Genome size is 5,076,418 bp and G+C content is 65.44%. A total of 4,096 protein-coding genes and 92 RNAs are predicted.

## ANNOUNCEMENT

*Xanthomonas arboricola* pv. pruni (*Xap*) is the causal agent of bacterial spots of stone fruits and almonds (1). The disease is currently distributed across almost all stone fruit-producing countries and mainly affects peach, nectarine, apricot, and Japanese plum production (2). In Italy, it has been reported on cherry laurel (3), peach, plum (4), and more recently on almond (5).

We report the draft genome sequence of *Xap* strain PVCT 262.1, isolated from almond leaves exhibiting bacterial spot symptoms collected in a commercial orchard in the Agrigento province, Sicily, Italy [Global Positioning System (GPS) coordinates, 37.290807°N, 13.584432°E] in 2020.

For *Xap* isolation, leaf tissue (2 mm) from lesion margins was crushed with sterile water. The suspensions were spread onto Yeast-Peptone-Glucose Agar (YPGA). Plates were incubated at 28°C for 2–3 days.

*Xap* was identified using conventional duplex PCR with specific primers (*Xarb*Q-F/*Xarb*Q-R and *Xap*Y17-F/*Xap*Y17-R) (6), *rpoD* gene partial sequencing (99.63% identity with *Xap* 15-088, GenBank accession number CP044334.1), and pathogenicity tests on 1-year-old grafted almond plants and detached leaves (7). For total DNA extraction and whole-genome sequencing, a single bacterial colony was transferred from YPGA to lysogenic broth (LB) and incubated overnight (180 rpm, 28°C). DNA was extracted from 1 mL culture (1 × 10^9^ cfu mL^−1^) with Gentra Puregene bacterial DNA extraction kit (Qiagen, Hilden, Germany), following the manufacturer’s instructions.

Sequencing libraries were constructed at Beijing Novogene Bioinformatics Technology Co., Ltd using Illumina NEBNext Ultra DNA Library Prep Kit (NEB, USA; 350 bp insert size), according to the manufacturer’s recommendations. Sequencing was performed on Illumina NovaSeq 6000 platform (sequencing depth, 312×; paired-end read length, 150  bp). Raw reads (14,580,000 bp) were demultiplexed, recorded in FASTQ file using bcl2fastq v2.19 and quality filtered with Fastp v0.23.1 (8), obtaining 11,366,667 bp clean reads (*Q*_30_ = 94.62%).

Reads were assembled using SOAPdenovo v2.04 (9), SPAdes v3.11.1 (10), Abyss v1.5.2 (11), and CISA v1.3 (12) for integration. Gaps were solved with SOAP GapCloser v1.12 (9). The annotation was performed on the NCBI Prokaryotic Genome Annotation Pipeline (PGAP) v6.6 (13). Default parameters were used except where otherwise noted.

The draft genome assembly generated 60 scaffolds (>500 bp) with 5,076,418 bp total length (genome coverage, 99.99×; *N*_50_ length, 165,685  bp) and 65.44% GC content. Genome completeness was estimated at 99.8% using BUSCO v5.6.1 (14) and xanthomonadales_odb10 (2024-01-08) lineage. PGAP predicted 4,096 protein-coding genes, 188 pseudogenes, and 92 RNAs.

PVCT 262.1 genome harbors copper resistance genes (e.g., *copB*, *copD-* and *copL*-family, *cutA*, *cutC*), suggesting copper resistance acquisition. The latter was observed *in vitro* for Italian *Xap* isolates, although *copLAB* gene cluster was not detected, suggesting different copper resistance mechanisms (4). The type III effector gene *xopE3*, specific to pathovar pruni within *X. arboricola* (15), was identified.

The genome sequence provided here will help further studies on bacterial spot ecology and epidemiology, contributing to elucidate almond-*Xap* interactions.

## Data Availability

This Whole-Genome Shotgun project was deposited in GenBank under the accession number JAZHEZ000000000, BioProject accession number PRJNA1061488, BioSample accession number SAMN39271251. The version described is JAZHEZ010000000. SRA accession number is SRR27587392.
